# Clinical Utility of Ultra-Widefield Fundus Photography with SS-OCT Images in Justifying Prophylactic Laser Photocoagulation of Peripheral Retinal Lesions

**DOI:** 10.3390/bioengineering12121367

**Published:** 2025-12-16

**Authors:** Joanna Żuk, Krzysztof Safranow, Anna Machalińska

**Affiliations:** 1 First Department of Ophthalmology, Pomeranian Medical University, Al. Powstańców Wielkopolskich 72, 70-111 Szczecin, Poland; joasia.zuk@gmail.com; 2 Department of Biochemistry and Medical Chemistry, Pomeranian Medical University, Al. Powstańców Wielkopolskich 72, 70-111 Szczecin, Poland; chrissaf@mp.pl

**Keywords:** optical coherence tomography (OCT), swept-source OCT (SS-OCT), ultra-widefield (UWF) imaging, peripheral retinal lesions, peripheral vitreoretinal interface, lattice degeneration (LD), snail-track degeneration, horseshoe retinal tear, prophylactic laser therapy

## Abstract

We aimed to validate the feasibility of combining ultra-widefield (UWF) fundus photography with targeted swept-source optical coherence tomography (SS-OCT) for clinical decision-making regarding a prophylactic laser therapy. For this purpose we enrolled 119 patients (135 eyes) who, basis on fundus examination, were eligible for prophylactic photocoagulation of degenerative retinal lesions. Eyes were classified into two groups: (1) justified laser, when SS-OCT confirmed vitreoretinal traction and/or subretinal fluid beneath the neurosensory retina; and (2) non-justified laser, when SS-OCT did not confirm these criteria. Using this SS-OCT-guided UWF approach, we found that 25.1% of eyes that initially qualified for laser based on clinical examination did not meet the SS-OCT criteria. Patients in the justified laser group were significantly younger than those in the non-justified group. Horseshoe retinal tears, lattice degeneration and snail-track degenerations, multiple lesions, and lesions located in the far and mid-periphery were significantly more frequent in the justified laser group than in the non-justified group. By contrast, the prevalence of operculated holes, bilateral lesions, and degenerative lesions in patients with a retinal detachment in the fellow eye did not differ between groups. Our findings suggest the SS-OCT-guided UWF imaging may refine patient selection for prophylactic laser therapy.

## 1. Introduction

Degenerative changes in the peripheral retina represent a diverse group of lesions that vary in morphology, anatomical depth, and clinical significance. Traditionally, peripheral retinal lesions have been categorized according to their propensity to cause rhegmatogenous retinal detachment (RRD) and their anatomical localization within the retinal layers. On the basis of anatomical depth, three primary types of lesions can be distinguished: intraretinal, vitreoretinal, and chorioretinal [1].

A traditional framework classifies peripheral retinal lesions by their risk of RRD, separating relatively benign entities from those that confer higher risk [1]. Lesions that may be predispose to retinal tears and RRD include lattice degeneration (LD), degenerative retinoschisis, cystic retinal tufts, and, more rarely, zonular traction tufts. Accordingly, these lesions are often considered for prophylactic therapy [2]. In this context, RRD—a major cause of vision loss—is largely preventable. While many of these lesions remain asymptomatic and are discovered incidentally, they become clinically significant when coupled with risk factors such as high myopia (with increased axial length) and active posterior vitreous detachment (PVD). Indeed, at least half of untreated symptomatic retinal breaks with persistent vitreoretinal traction (horseshoe or flap tears) will lead to clinical RRD unless treatment is applied [3]. The goal of treatment for retinal breaks and lesions is to create chorioretinal adhesion surrounding the retinal tear using laser photocoagulation, thereby halting the progression of subretinal fluid and detachment of the neurosensory retina. Laser barrage around these symptomatic tears reduces the risk of RRD to less than 5% [3]. However, there is no consensus regarding treatment and insufficient evidence to guide the management of asymptomatic retinal breaks and holes. Given the marked heterogeneity of degenerative peripheral retinal changes that may lead to rhegmatogenous retinal detachment (RRD), improved diagnostic triage and treatment selection are needed to prevent avoidable RRD [1,2,3,4]. There are no randomized controlled trials to support treatment protocols for asymptomatic retinal holes and lesions to prevent retinal detachment [4]. Abnormal peripheral vitreoretinal adhesion and traction appear to be important factor that may pull the adjacent retina, leading to a tear and subsequent RRD [3,5]. To date, evaluations of vitreous status at the site of peripheral retinal lesions with conventional clinical examination or fundus photography have been limited. Ultra-widefield (UWF) retinal imaging has enabled the visualization of peripheral retinal lesions and, along with integrated swept-source optical coherence tomography (SS-OCT), has provided new insights into vitreous and retinal interactions at the site of peripheral pathology. Visualizing the retinal morphology and vitreoretinal interface associated with peripheral lesions on SS-OCT might allow the treating clinician to make better-informed decisions. A detailed analysis of the structural characteristics of peripheral lesions, such as vitreous traction or peripheral subretinal fluid at the edge of the lesion, may aid clinicians in determining whether laser intervention is necessary.

Thus, we aimed to validate the feasibility of using SS-OCT imaging for clinical decision-making with respect to laser intervention. Accordingly, the objective of the study was to characterize the morphological status of the lesions on based on SS-OCT and to determine the proportion of cases in which peripheral retinal laser photocoagulation was justified.

## 2. Materials and Methods

This consecutive case series included 119 patients (135 eyes) eligible for prophylactic laser photocoagulation of peripheral lesions at the 1st Department of Ophthalmology, Pomeranian Medical University, Szczecin, Poland from January 2024 to August 2025. Initial fundus-based qualification for retinal laser therapy was made by two ophthalmologists, one of whom was a retinal specialist experienced in laser treatment. Each examiner made an independent judgment based on a dilated fundus examination using indirect ophthalmoscopy with the Volk Super Quad 160 lens. All cases included in the analysis were classified by both examiners as candidates for laser treatment; the initial qualification therefore reflects concordant interpretations. Inclusion criteria for photocoagulation were as follows: (i) the presence of horseshoe retinal tears, operculated holes, or other retinal breaks associated with recent-onset floaters; (ii) atrophic holes or lattice degeneration in eyes whose fellow eye had previously developed rhegmatogenous retinal detachment (RRD); and (iii) peripheral degenerative lesions in eyes with marked degenerative myopia and/or vitreous condensations overlying the lesion.

**Figure 1:**
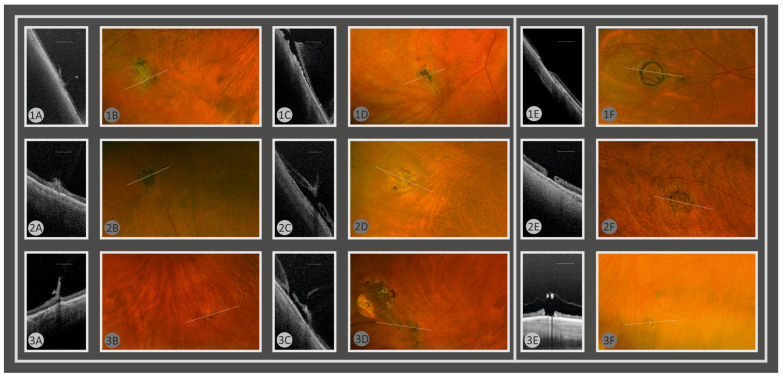
Representative SS-OCT (**A**,**C**,**E**) with corresponding ultra-widefield (UWF) color fundus images (**B**,**D**,**F**) acquired at the same peripheral lesion sites. Numerals 1–3 denote the three example cases (1 = A–B; 2 = C–D; 3 = E–F). These cases illustrate lesions that did or did not meet the SS-OCT criteria for a justified prophylactic laser indication. A laser indication was considered justified when SS-OCT demonstrated (i) peripheral subretinal fluid at the lesion site and/or (ii) focal vitreoretinal traction. The yellow line in panels B, D, and F indicates the OCT scan location. Scale bar in panels A, C, and E represents 1000 µm. (**A**) SS-OCT (case 1, 2 and 3): focal vitreoretinal traction at the lesion edge—meets SS-OCT criteria (justified). (**B**) UWF (case 1, 2 and 3): corresponding peripheral lesion; white line shows scan location. (**C**) SS-OCT (case 1, 2 and 3): subretinal fluid associated with vitreoretinal traction—meets SS-OCT criteria (justified). (**D**) UWF (case 1, 2 and 3): corresponding peripheral lesion; white line shows scan location. (**E**) SS-OCT (case 1, 2 and 3): no vitreoretinal traction and no subretinal fluid—does not meet SS-OCT criteria (not justified). (**F**) UWF (case 1, 2 and 3): corresponding peripheral lesion; white line shows scan location.

The qualification decisions were subsequently verified by UWF fundus photography with targeted SS-OCT. An indication for prophylactic laser was considered justified when SS-OCT demonstrated either (1) peripheral subretinal fluid at the lesion site and/or (2) focal vitreoretinal traction at the lesion site (Figure 1).

Discrepancies between the initial clinical qualification and the SS-OCT assessment reflect the greater sensitivity and spatial resolution of UWF-guided SS-OCT for peripheral degenerative lesions. By contrast, on indirect ophthalmoscopy, shallow neurosensory retinal elevation, subretinal fluid, subtle focal vitreoretinal traction can be difficult to detect, whereas these features are readily visualized with SS-OCT at the lesion site. Consequently, SS-OCT sometimes identified these features when they were not appreciated clinically and, in other instances, confirmed their absence when suspected.

UWF fundus images were captured on an Optos Silverstone P200Txe Ultra-Widefield Imaging Device with SS-OCT (Dunfermline, UK). Technical specifications: SS-OCT at 1050 nm, A-scan rate up to 100 kHz; axial resolution < 7 (in tissue); transverse resolution < 20 µm (in tissue); scan depth up to 2.5 mm; Class 1 laser safety standards [6,7]. OCT volume scans (121 B-scans covering an area of 6 mm × 6 mm) or high-density volume scans (121 B-scans covering an area of 3.5 mm × 6 mm) were used, with pattern choice determined by lesion size and imaging goal: 6 × 6 mm for broader coverage of the lesion and margins, and 3.5 × 6 mm high-density when greater vitreoretinal-interface detail was required.

To optimize peripheral imaging, all eyes were pharmacologically dilated; lesions were localized on the scanning laser ophthalmoscopy (SLO) image, and an OCT scan was used to steer and center the raster on the lesion with real-time retinal registration/beam-steering. We centered the lesion within the scan, repeated acquisitions when artifacts were present, and excluded very anterior lesion or insufficient-quality scans.

Imaging was performed by trained ophthalmic technicians using a standardized UWF-guided, targeted SS-OCT workflow. Before the examination, all patients were pharmacologically dilated to optimize peripheral visualization and reduce vignetting.

SS-OCT analyses were performed only in eyes with clear media following pharmacologic dilation. Exclusion criteria included media haze (secondary to corneal opacity, cataract, or vitreous opacities) and insufficient image quality. Scans were included only if the signal was adequate, the lesion was centered, and there were no limiting artifacts (e.g., severe motion, segmentation failure, eyelid or eyelash shadowing, or marked decentration). When adequate imaging could not be achieved or the lesion was too peripheral, the eye was excluded from the SS-OCT-based analysis.

Descriptive statistics (mean ± standard deviation (SD), %) were used to summarize the study data. Differences in age between groups were assessed with the Mann-Whitney U test. For categorical variables, two-way (2 × 2) contingency tables were constructed and two-tailed Fisher’s exact test was used. A two-sided *p* value < 0.05 was considered statistically significant. Statistical analyses were performed using Statistica 13 software. Effect sizes are reported as odds ratios (OR) with 95% confidence intervals (95% CI). No corrections for multiple comparisons were applied (exploratory analysis).

## 3. Results

A total of 135 eyes from 119 patients aged between 11 and 83 years, comprising 54 females and 65 males, were included in the study. The mean age of the patients was 61.82 years. On initial clinical examination, retinal holes were detected in 48.15% of the lesions; 19.26% were horseshoe tears, and 17.04% were operculated holes. LD was observed in 25.19% of eyes, and snail-track degeneration was observed in 10.37% of eyes. In 27.41% of patients, degenerative changes were bilateral. Degenerative changes were identified as solitary in 54.07% of cases, whereas in 45.93% multiple lesions were present. Degenerative changes were observed in 89.63% of the cases on the far periphery and in 16.30% of cases on the mid-periphery of the eye fundus. RRD in the fellow eye was documented in 10.37% of patients.

SS-OCT scans demonstrated vitreoretinal traction adjacent to the retinal lesion in 65.93% of cases, neurosensory retinal elevation in 42.22% of cases, and the presence of intraretinal cysts in 27.41% of cases. Retinal atrophy and thinning corresponding to the location of the degenerative changes were present in 46.67% of cases, whereas retinoschisis was present in 25.19% of cases. Retinal breaks were observed in 59.26% of cases.

Furthermore, we divided the study population into two groups based on the SS-OCT findings: (1) the justified laser group, which met the SS-OCT criteria for prophylactic photocoagulation, defined as the presence of subretinal fluid and/or evident vitreoretinal traction at the lesion site (n = 101; 74.9% of all eyes), and (2) the non-justified laser group, which was initially eligible for laser coagulation on the basis of clinical examination but did not fulfill the SS-OCT criteria (n = 34; 25.1% of all eyes). The clinical characteristics of the two groups are summarized in Table 1.

Patients in the justified laser group were significantly younger than those in the non-justified group (*p* = 0.001). Both far-periphery (*p* = 0.045, OR [95% Cl] = 3.48 (1.11–10.91)) and mid-periphery (*p* = 0.029, OR [95% Cl] = 0.32 (0.12–0.85)) lesions were significantly more frequent in the justified laser group than in the non-justified group. Multiple degenerative changes (*p* = 0.003, OR [95% Cl] = 3.73 (1.53–9.11)) and horseshoe retinal tears (*p* = 0.005, OR [95% Cl] = 10.86 (1.38–85.08)) were significantly more frequent in the justified laser group than in the non-justified group. Interestingly, no significant difference was observed in the prevalence of operculated retinal holes between the justified and non-justified laser groups (*p* = 0.292, OR [95% Cl] = 0.57 (0.21–1.50)). Similarly, the incidence bilateral degenerative changes did not differ significantly between groups (*p* = 0.075, OR [95% Cl] = 2.69 (0.94–7.66)). Among the degenerative lesions LD and snail-track degeneration were significantly more frequent in the justified laser group than in the non-justified group (*p* < 0.001 OR [95% Cl] = 16.01 (2.06–124.56) and *p* = 0.021, respectively, OR ∞ (-)). The presence of RRD in the fellow eye was not justified by laser treatment in this cohort (*p* = 0.517, OR [95% Cl] = 2.16 (0.45–10.32)).

## 4. Discussion

To date, there are no clear guidelines for the laser treatment of peripheral retinal lesions. An attempt to systematize indications was made in 2019 and updated in 2024 by the American Academy of Ophthalmology (AAO) [3,8]. However, the proposed directives remain ambiguous and do not strictly define recommendations for preventive treatment. Indeed, traditional evaluation of the peripheral retina via dilated fundus examination or UWF color photography provides limited cross-sectional detail regarding vitreoretinal relationships and microstructural changes. UWF fundus photography with SS-OCT helps overcomes these limitations by offering high-resolution, wide-field visualization of peripheral retinal architecture and the vitreous interface. This enhanced imaging capability improves the detection, characterization, and understanding of peripheral degenerative lesions, offering new insights into management strategies. However, UWF imaging with SS-OCT is also subjected to optical limitations, including local defocus and shadowing, curvature-related tilt, and susceptibility to segmentation failures and motion or blink artifacts. These factors can reduce sensitivity, particularly in very anterior (ora-adjacent) lesions. To the best of our knowledge, no standardized OCT-based criteria for prophylactic photocoagulation of peripheral lesions have been established to date. Recently, to identify patients who may benefit from retinal photocoagulation, Zheng et al. [9] proposed a contemporary OCT-based classification of peripheral vitreoretinal interface lesions reflecting their progression risk. They differentiated categorized A lesions as lacking vitreoretinal traction, category B1 lesions as having evident vitreoretinal traction, category B2 lesions as having both significant traction and cystic intraretinal changes and category C as retinal breaks with an elevated edge representing true retinal tears [9]. Thus, we presumed that key indicators of lesion progression include the presence of focal vitreoretinal traction and/or peripheral subretinal fluid. To our knowledge, no published study has demonstrated a linear dose-response relationship between the magnitude of neurosensory elevation and tear risk [10]. We found that approximately one quarter of patients who initially qualified for laser coagulation on the basis of clinical fundus examination did not meet the SS-OCT criteria for prophylactic treatment. To the best of our knowledge, this is the first study to assess the proportion of SS-OCT-guided, justified versus non-justified prophylactic laser treatments in peripheral degeneration.

In accordance with the American Academy of Ophthalmology (AAO) recommendations, acute symptomatic horseshoe tears and retinal dialyses should be treated promptly based on of existing evidence [3]. This is consistent with our findings, which demonstrate that horseshoe tears were significantly more frequent in the justified-laser group than in the non-justified group. According to the literature, at least half of untreated symptomatic retinal breaks with persistent vitreoretinal traction (such as horseshoe or flap tears) will lead to clinical retinal detachment if left untreated [3]. Prompt laser photocoagulation to create a chorioretinal adhesion around these symptomatic tears reduces the risk of retinal detachment to less than 5% [3]. In contrast, laser treatment for operculated retinal holes in asymptomatic patients is rarely recommended [3]. Even when symptoms are present, treatment may not be necessary [3]. This aligns with our findings, which showed no significant difference in the prevalence of operculated retinal holes between the justified and non-justified laser group. Operculated retinal breaks are less frequently associated with retinal detachment when evaluated by ophthalmoscopy, likely because of the absence of residual vitreoretinal traction [11,12]. Using UWF fundus photography with SS-OCT [13], Govetto et al. recently demonstrated that the morphology of operculated round holes was similar to that of stage 4 full-thickness macular holes with no vitreous traction. Nevertheless, retinal detachment secondary to operculated breaks can occasionally occur [3,11,14].

Importantly, we found that patients in the justified laser group were significantly younger than those in the non-justified group. Indeed, PVD is an age-dependent process [15]. Once the detachment is complete, the risk of vitreoretinal traction and subsequent retinal tearing markedly decreases [16,17,18]. The strongest evidence linking younger age to an increased risk of retinal tears in the context of PVD comes from Jindachomthong et al. [16], who reported that patients younger than 60 years with acute symptomatic PVD had a greater risk of delayed retinal tears. Similarly, a large prospective study by Nixon, Davie, and Snead [19] revealed that approximately 9.9% of eyes with PVD presented with retinal tears or detachments at the time of diagnosis. Furthermore, Cox-Mantel log-rank analysis [16] revealed that patients who were younger (<60 years), myopic, or had lattice degeneration were more likely to develop retinal tears.

LD is present in approximately 6-8% of the general population and in nearly 30% of phakic retinal detachment cases [19,20]. According to the AAO recommendations, asymptomatic lattice retinal degeneration with holes usually does not require treatment [3]. However, our results strongly indicate that cases of LD met the SS-OCT criteria for prophylactic photocoagulation and were significantly more frequent in the justified laser group than in the non-justified group. Indeed, vitreoretinal adhesion within areas of lattice degeneration is believed to be responsible for the majority of lattice-related retinal detachments [21]. The creation of a chorioretinal adhesion surrounding retinal breaks or areas of LD by laser photocoagulation has long been advocated as an effective preventive measure against retinal detachment [4]. However, some current treatment recommendations, although based on expert consensus, are not consistently supported by high-level evidence [4]. Consequently, considering that snail-track degeneration may represent a variant or early form of lattice degeneration, there are no specific recommendations regarding the management of this type of lesion [22]. Our data clearly show that snail-track degenerations were significantly more frequent in the justified-laser group than in the non-justified group. Accordingly, the risk of retinal detachment due to an asymptomatic retinal break in individuals without detachment in the fellow eye was approximately 0.5% over a follow-up period averaging 11 years [4,23]. In accordance with the AAO recommendations, there is no consensus on treatment and insufficient evidence to guide management in eyes with atrophic holes or lattice retinal degeneration where the fellow eye has had an RRD [3]. Our data indicate that the presence of RD in the fellow eye did not differ between the justified and the non-justified laser group. Similarly, the incidence of bilateral degenerative changes did not differ significantly between groups. Given this variability, an objective and reproducible method to determine which patients would truly benefit from prophylactic laser treatment is clearly needed. In this context, SS-OCT may provide valuable structural information to guide more evidence-based decision-making. Interestingly, in a study by Adina D. Kazan et al. [17], the authors identified multiple retinal defects without detachment in the fellow eye as one of the high-risk features for RRD [17]. These findings are consistent with our data demonstrating that the presence of multiple peripheral lesions were significantly more frequent in the justified-laser group than in the non-justified group and supported laser treatment in the majority of cases.

## 5. Conclusions

In summary, conventional fundus examination does not always permit definitive characterization of lesions or support informed clinical decision-making regarding prophylactic laser photocoagulation. The use of UWF fundus photography combined with targeted SS-OCT enables more accurate assessment of peripheral retinal changes and may reduce unnecessary prophylactic laser procedures. Thus, the novelty of our study lies not in the imaging hardware, but in the implementation of a reproducible, SS-OCT-driven qualification algorithm for prophylactic laser photocoagulation that rationalizes patient selection and identifies a subset of eyes in which treatment may be unwarranted [9,11,13,24]. In the longer term, recent computer-vision advances and methodological directions in deep learning [25,26,27] give a promise to furnish more precise functional prognoses in this area that may help to refine and standardize the qualification criteria for prophylactic treatment.

## Figures and Tables

**Table 1:** Baseline clinical and lesion characteristics of eyes referred for prophylactic laser, stratified by SS-OCT—adjudicated indication (Justified vs. Non-justified).

Clinical Characteristics	Justified Laser Therapy	Non-Justified Laser Therapy	OR (95% Cl)	*p*
Number of eyes: n	101	34		
Age (average ± SD)	51.38 ± 16.59	61.82 ± 14.67		**0.001**
Sex F/M n (%)	42/59 (41.58/58.42)	16/18 (47.06/52.94)	1.25 (0.57–2.75)	0.689
Horseshoe tears (%)	24.75	2.94	10.86 (1.38–85.08)	**0.005**
Operculated holes (%)	14.85	23.53	0.57 (0.21–1.50)	0.292
Lattice retinal degeneration (%)	32.67	2.94	16.01 (2.06–124.56)	**<0.001**
Snail track degeneration (%)	13.86	0.00	∞ (-)	**0.021**
Multiple degenerative changes (%)	53.47	23.53	3.73 (1.53–9.11)	**0.003**
Bilateral lesions (%)	31.68	14.71	2.69 (0.94–7.66)	0.075
Far-peripheral location (%)	93.07	79.41	3.48 (1.11–10.91)	**0.045**
Mid-peripheral location (%)	11.88	29.41	0.32 (0.12–0.85)	**0.029**
Documented RRD in the fellow eye (%)	11.88	5.88	2.16 (0.45–10.32)	0.517

Values are n (%) unless stated otherwise. Age is mean ± SD (Mann-Whitney U). For categorical variables, odds ratios (OR) with 95% confidence intervals (95% CI) are from 2 × 2 Fisher’s exact tests; two-sided *p*-values are reported to three decimal places, with *p* < 0.001 shown as “<0.001”; bold *p*-values indicate statistical significance (*p* ≤ 0.05). Far-peripheral and mid-peripheral location refer to clinician-recorded lesion position on UWF imaging. RRD = rhegmatogenous retinal detachment.

## Data Availability

The data that were used to support the findings of this study are available from the corresponding author upon request.

## Abbreviations

The following abbreviations are used in this manuscript:

UWF	ultra-widefield
SS-OCT	swept-source optical coherence tomography
LD	lattice degeneration
RRD	rhegmatogenous retinal detachment
AAO	American Academy of Ophthalmology
PVD	posterior vitreous detachment
SLO	Scanning Laser Ophthalmoscopy
